# Adults at high-risk of severe coronavirus disease-2019 (Covid-19) in Brazil

**DOI:** 10.11606/s1518-8787.2020054002596

**Published:** 2020-05-15

**Authors:** Leandro F. M. Rezende, Beatriz Thome, Mariana Cabral Schveitzer, Paulo Roberto Borges de Souza-Júnior, Célia Landmann Szwarcwald

**Affiliations:** I Universidade Federal de São Paulo Escola Paulista de Medicina Departamento de Medicina Preventiva São PauloSP Brasil Universidade Federal de São Paulo. Escola Paulista de Medicina. Departamento de Medicina Preventiva. São Paulo, SP, Brasil; II Fundação Instituto Oswaldo Cruz Instituto de Comunicação e Informação Científica e Tecnológica em Saúde Rio de JaneiroRJ Brasil Fundação Instituto Oswaldo Cruz. Instituto de Comunicação e Informação Científica e Tecnológica em Saúde. Rio de Janeiro, RJ, Brasil

**Keywords:** Risk Groups, Coronavirus Infections, epidemiology, Socioeconomic Factors, Patient Care Planning

## Abstract

**OBJECTIVE:**

To estimate the proportion and total number of the general adult population who may be at higher risk of severe Covid-19 in Brazil.

**METHODS:**

We included 51,770 participants from a nationally representative, household-based health survey (PNS) conducted in Brazil. We estimated the proportion and number of adults (≥ 18 years) at risk of severe Covid-19 by sex, educational level, race/ethnicity, and state based on the presence of one or more of the following risk factors: age ≥ 65 years or medical diagnosis of cardiovascular disease, diabetes, hypertension, chronic respiratory disease, cancer, stroke, chronic kidney disease and moderate to severe asthma, smoking status, and obesity.

**RESULTS:**

Adults at risk of severe Covid-19 in Brazil varied from 34.0% (53 million) to 54.5% (86 million) nationwide. Less-educated adults present a 2-fold higher prevalence of risk factors compared to university graduated. We found no differences by sex and race/ethnicity. São Paulo, Rio de Janeiro, Minas Gerais, and Rio Grande do Sul were the most vulnerable states in absolute and relative terms of adults at risk.

**CONCLUSIONS:**

Proportion and total number of adults at risk of severe Covid-19 are high in Brazil, with wide variation across states and adult subgroups. These findings should be considered while designing and implementing prevention measures in Brazil. We argue that these results support broad social isolation measures, particularly when testing capacity for SARS-CoV-2 is limited.

## INTRODUCTION

The World Health Organization (WHO) suggests that most people infected with the virus may develop mild or uncomplicated (80%) coronavirus disease 2019 (Covid-19), while the remaining 20% may develop its severe variation, requiring hospitalization (14%) or intensive care unit (6%)^1^. Established risk factors for severe disease among inpatients with Covid-19 in China included older age^2,3^ and serious medical conditions such as cardiovascular disease^2^, diabetes^2^, chronic respiratory disease (in particular chronic obstructive pulmonary disease – COPD)^2^, hypertension^2,4^, cancer^2,5^, and cerebrovascular disease^3,4^. Recent findings from United States (US) and Europe confirmed these risk factors and proposed new ones, such as chronic kidney disease, obesity, asthma and smoking^6^.

The emergence of a highly transmissible pathogen^10^ in a completely susceptible population has resulted in an exponential growth of new cases worldwide and a wide dissemination across the globe. As of April 12, 2020, the number of SARS-CoV-2 infections was above 1.8 million, reported in 185 countries/regions of the world^11^. High- and low-income regions are already facing overload of health facilities and facing scarcity of resources to fight the pandemic. In lower resource settings, countries have a short time to prepare prevention and management strategies, including the identification of high-risk populations and regions within countries.

Herein, we propose a calculation of the proportion and total number of the general adult population who may be at higher risk for severe Covid-19, based on routinely collected data from a nationwide, household-based survey in Brazil. We argue that this method could be easily and rapidly applied within and across countries in order to craft tailored prevention strategies such as social isolation.

## METHODS

We obtained data from the most recent representative, household-based survey conducted in Brazil, the National Health Survey (PNS, 2013 – *Pesquisa Nacional de Saúde*), carried out by the Ministry of Health in partnership with the Brazilian Institute of Geography and Statistics (IBGE). The PNS enrolled 62,202 adults who responded to a comprehensive questionnaire about several health-related issues. In this study, we included 51,770 participants who responded to the questionnaire about medical diagnosis and lifestyle risk factors, and had their weight and height measured. Further details about PNS have been described elsewhere^12^.

### Risk Factors for Severe Covid-19

We included risk factors for severe Covid-19 based on currently available information from clinical studies and expertise^2^, and for which exposure data were available in the PNS^12^. Age and medical diagnosis of cardiovascular disease, diabetes, hypertension, chronic respiratory disease, cancer, stroke, chronic kidney disease and asthma were assessed. We also obtained time (in years) since cancer diagnosis and treatment/medication use for chronic kidney disease (e.g. dialysis) and asthma to match definitions from the literature (e.g. moderate to severe asthma). Information about age, smoking status and measured body mass index (BMI) were also obtained/estimated.

Prevalence of one or more risk factors for severe Covid-19 was estimated using two criteria (Table 1). Criterion 1 included first identified and established risk factors for severe Covid-19 such as age ≥ 65 years or medical diagnosis of cardiovascular disease, diabetes, hypertension, chronic respiratory disease, cancer or stroke. Although ≥ 60 years have been used to define older adults in Brazil, herein we considered ≥ 65 years to match the definition of risk factors for Covid-19 obtained from the literature and allow comparisons with other publications^2^. Criterion 2 additionally included diagnosis of chronic kidney disease and moderate to severe asthma, smoking status (current smokers) and obesity (BMI ≥ 30 kg/m^2^). Criterion 2 was used to provide a higher sensitivity for the proportion of adults at risk of severe illness. Denominator for both criteria 1 (n = 52,511) and 2 (n = 51,770) included all participants with complete questionnaires. We also estimated the sum of all risk factors for severe illness (0, 1, 2, 3 + risk factors).

**Table 1:** Definition of risk factors for severe Covid-19 according to two different proposed criteria.

Risk factors	Definition	Presence of risk factor for severe Covid-19	

		Criterion 1	Criterion 2
Age	in years	≥ 65 years	≥ 65 years
Cardiovascular disease	Has a doctor ever diagnosed you with a heart disease such as infarction, angina, heart failure or other?	Yes	Yes
Diabetes	Has a doctor ever diagnosed you with diabetes?	Yes	Yes
Hypertension	Has a doctor ever diagnosed you with hypertension (high blood pressure)?	Yes	Yes
Chronic respiratory disease	Has a doctor already diagnosed you with any lung disease such as pulmonary emphysema, chronic bronchitis, or COPD (Obstructive Pulmonary Disease Chronic)?	Yes	Yes
Cancer	Has any doctor ever diagnosed you with cancer (excluding skin cancer)?	Yes	Yes
	How many years ago since your cancer diagnosis?	< 5 years	< 5 years
Stroke	Has any doctor ever diagnosed you with stroke?	Yes	Yes
Obesity	Measured body mass index	No	≥ 30 kg/m^2^
Smoking	Current smoker	No	Yes (daily or less than daily)
Chronic kidney disease	Has any doctor ever diagnosed you with chronic kidney disease?	No	Yes
	What do you currently do or have done because of the chronic kidney disease?	No	Hemodialysis, peritoneal dialysis, took medication, underwent a kidney transplant
Moderate to severe asthma	Has any doctor ever diagnosed you with asthma (or asthmatic bronchitis)?	No	Yes
	What do you currently do because of asthma?	No	Use of inhalers, aerosol or tablets

### Sociodemographic Covariates

Information on covariates including sex, race/ethnicity, educational level, and Brazilian state (26 states and the Federative District) were obtained to describe the proportion of adults at risk of severe Covid-19 by population strata. We also retrieved the total projected number of the Brazilian adult population (≥ 18 years) in 2020 by sex and state from the IBGE^13^.

### Statistical Analysis

We estimated the prevalence and 95% confidence intervals of adults at risk for severe Covid-19 (Criterion 1 and Criterion 2) by sex, education, race/ethnicity and Brazilian state. We performed sensitivity analyses for prevalence by considering two other definitions for older adults (≥ 60 years and ≥ 70 years). In order to obtain the total number of adults at risk of severe illness, we applied the prevalence to the number of adult’s population (≥ 18 years) by sex and state. The sample design was considered for all analyses using the survey prefix command (svy) in Stata version 15.0.

## RESULTS

Participants characteristics and risk factors for severe illness are presented by age group (Table 2). Compared with younger participants, older adults (≥ 65 years) were less educated, more likely women, white and presented higher prevalence of risk factors for severe Covid-19, except for smoking. Prevalence of one or more risk factors for severe illness was 47.3% in younger vs 75.9% in older adults.

**Table 2:** Characteristics and risk factors for severe Covid-19 by age group in Brazil, PNS 2013

Characteristics	Age groups		Total

	< 65 years	≥ 65 years	
**Number of participants**	23.838	27.932	51.770
**Mean age, years (se)**	39.7 (11.4)	73.5 (14.1)	44.3 (15.0)
**Sex (%)**			
Men	45.4	42.9	45.0
**Education (%)**			
None or incomplete primary education	15.1	67.0	22.2
Complete primary or incomplete secondary education	27.2	14.0	25.4
Complete secondary education or incomplete undergraduate course	42.7	10.3	38.3
University Graduate	15.0	8.7	14.1
**Race/ethnicity (%)**			
White	48.3	55.9	49.4
Non-white	51.7	44.1	50.6
**Risk factors for Severe Covid-19 (%)**			
Cardiovascular disease	3.4	13.0	4.7
Diabetes	5.1	20.7	7.2
Chronic respiratory disease	1.5	4.4	1.9
Hypertension	18.8	55.3	23.7
Cancer	0.6	2.2	0.8
Stroke	1.0	6.1	1.7
Obesity (BMI ≥30 kg/m^2^)	22.0	22.7	22.1
Smoking	14.6	9.6	13.9
Chronic kidney disease	0.7	2.0	0.9
Moderate to severe asthma	1.5	1.7	1.5
Number of risk factors for severe Covid-19* (%)			
None	52.7	24.1	48.8
1	30.9	35.1	31.5
2	12.0	25.2	13.8
3+	4.4	15.6	5.9

SE: standard error

* Diagnosis of cardiovascular disease, diabetes, chronic respiratory disease, hypertension, cancer (< 5 years of diagnosis), stroke, obesity (BMI ≥ 30 kg/m^2^), current smoking, chronic kidney disease (diagnosis and under hemodialysis, peritoneal dialysis, taking medication or did a kidney transplant), moderate to severe asthma (diagnosis and taking inhalers, aerosol or tablets)

Proportion and total number of adults at risk for severe Covid-19 in Brazil varied from 34.0% (53 million adults) to 54.5% (86 million adults) (Table 3). Overall, 46% of the sample presented no risk factor, 30.0% with one, 15.0% with two, and 9% with 3 or more risk factors for severe illness. Sensitivity analyses considering older adults ≥ 60 years and ≥ 70 years suggested that prevalence could vary from 36.7%–56.2% to 32.3%–53.3%, respectively (Table 4).

**Table 3:** Prevalence of one or more risk factor for severe Covid-19 among the Brazilian general adult population by risk criteria and sociodemographic characteristics, PNS 2013.

Characteristics	Prevalence of one or more risk factors for severe Covid-19			

	Criterion 1 (n = 52,511)		Criterion 2 (n = 51,770)	
	
	Prevalence (%)	95%CI	Prevalence (%)	95%CI
**Total**	34.0	33.2–34.7	54.4	53.6–55.2
**Sex**				
Men	31.6	30.5–32.8	53.3	52.1–54.5
Women	35.9	34.9–36.8	55.4	54.3–56.4
**Education**				
None or incomplete primary education	66.3	64.7–67.9	80.2	78.9–81.4
Complete primary or incomplete secondary education	30.5	29.2–31.9	55.0	53.5–56.5
Complete secondary education or incomplete undergraduate course	20.4	19.4–21.4	42.2	40.9–43.6
University Graduate	27.0	25.1–29.1	46.1	44.1–48.3
**Race/ethnicity**				
White	34.9	33.8–36.0	55.0	53.9–56.2
Non-white	33.1	21.1–34.0	53.9	52.8–54.9

Criterion 1: age ≥ 65 years or diagnosis of cardiovascular disease, diabetes, chronic respiratory disease, hypertension, cancer (< 5 years of diagnosis), or stroke

Criterion 2: additionally, obesity (BMI ≥ 30 kg/m^2^), current smoking, chronic kidney disease (diagnosis and under Hemodialysis, peritoneal dialysis, taking medication or did a kidney transplant), moderate to severe asthma (diagnosis and taking inhalers, aerosol or tablets)

**Table 4:** Sensitivity analysis: prevalence of one or more risk factors for severe Covid-19 among the Brazilian general adult population by risk criteria, definitions of older age and sociodemographic characteristics in Brazil, PNS 2013.

Characteristics	Risk factors for severe Covid-19							

	Criterion 1 (n = 52,511)				Criterion 2 (n = 51,770)			
	
	Older age defined as ≥ 60 years		Older age defined as ≥ 70 years		Older age defined as ≥ 60 years		Older age defined as ≥ 70 years	
			
	Prevalence (%)	95%CI	Prevalence (%)	95%CI	Prevalence (%)	95%CI	Prevalence (%)	95%CI
**Total**	36.7	36.0–37.5	32.3	31.6–33.0	56.2	55.3–57.0	53.3	52.5–54.0
**Sex**								
Men	34.5	33.3–35.6	30.0	28.9–31.1	54.9	53.7–56.1	52.2	51.0–53.4
Women	38.6	37.6–39.5	34.2	33.3–35.1	57.2	56.1–58.2	54.2	53.2–55.2
**Education**								
None or incomplete primary	72.0	70.4–73.4	62.2	60.5–63.8	83.4	82.3–84.6	77.5	76.2–78.8
Complete primary or incomplete secondary	32.2	30.9–33.6	29.3	28.0–30.6	56.2	54.6–57.7	54.1	52.5–55.6
Complete secondary or incomplete university	22.1	21.1–23.2	19.8	18.8–20.8	43.4	42.0–44.7	41.8	40.4–43.2
University Graduate	30.0	28.0–32.1	25.6	23.7–27.5	48.1	45.9–50.2	45.1	43.0–47.2
**Race/ethnicity**								
White	38.0	36.9–39.2	33.1	32.0–34.2	57.0	55.8–58.2	53.8	52.6–54.9
Non-white	35.5	34.5–36.5	31.6	30.6–32.5	55.3	54.3–56.3	52.8	51.8–53.9

Criterion 1: age group or diagnosis of cardiovascular disease, diabetes, chronic respiratory disease, hypertension, cancer (< 5 years of diagnosis), or stroke; Criterion 2: additionally obesity (BMI ≥ 30 kg/m^2^), current smoking, chronic kidney disease (diagnosis and under hemodialysis, peritoneal dialysis, taking medication or did a kidney transplant), moderate to severe asthma (diagnosis and taking inhalers, aerosol or tablets).

Proportion of adults at risk for severe Covid-19 was 2-fold higher in less educated participants compared with university graduated. We found no differences in prevalence estimates by sex and race/ethnicity (Table 3). Estimates varied widely across states, with higher prevalence in the South and Southeast regions of the country (Figure). The highest prevalence was 39.5%–58.4% in Rio Grande do Sul, followed by 36.0–55.8% in Rio de Janeiro and 35.6%–58.2% in São Paulo. The lowest prevalence was found in Amapá (23.4%–45.9%), followed by Roraima (25.0%–48.6%) and Amazonas (25.1%–48.7%). The highest number of adults at risk of severe illness was found in São Paulo (17-21 million), Minas Gerais (6–9 million) and Rio de Janeiro (5–7 million) (Table 5).

**Figure:**
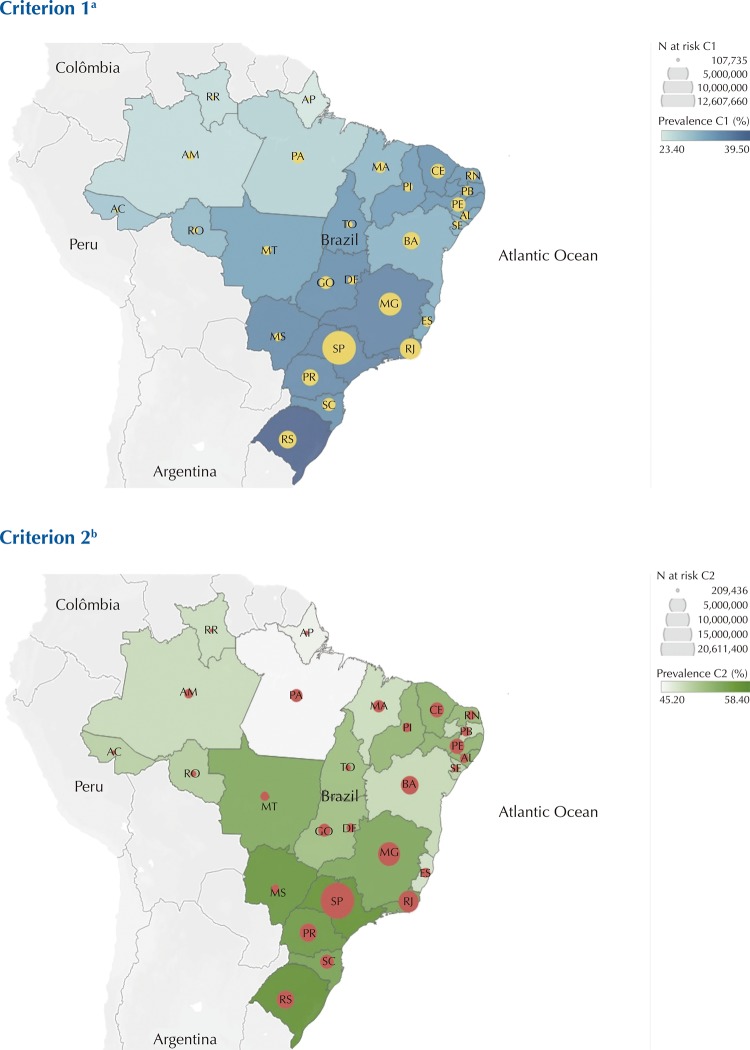
Adults at high-risk of severe Covid-19 in Brazil by state and risk criteria. ^a^ Criterion 1 (C1): age ≥ 65 years or diagnosis of cardiovascular disease, diabetes, chronic respiratory disease, hypertension, cancer (&lt;5 years of diagnosis), or stroke; ^b^ Criterion 2 (C2): additionally, obesity (BMI ≥ 30 kg/m^2^), current smoking, chronic kidney disease (diagnosis and under hemodialysis, peritoneal dialysis, taking medication or did a kidney transplant), moderate to severe asthma (diagnosis and taking inhalers, aerosol or tablets).

**Table 5:** Prevalence of one or more risk factors for severe Covid-19 among the Brazilian general adult population by risk criteria and Brazilian states, PNS 2013.

Brazilian States	Adult population (≥ 18 years)	Prevalence of one or more risk factors for severe Covid-19, %					

		Criterion 1 (n = 52,511)			Criterion 2 (n = 51,770)		
	
		Prevalence (%)	95%CI	N at risk	Prevalence (%)	95%CI	N at risk
Brazil	158,255,554	34.0	33.2–34.7	53,806,888	54.4	53.6–55.2	86,091,021
Brazilian States							
Rondônia	1,296,218	29.6	26.7–32.7	383,681	50.3	47.3–53.2	651,998
Acre	581,754	28.1	25.3–31.0	163,473	50.0	46.8–53.2	290,877
Amazonas	2,769,201	25.1	22.6–27.8	695,069	48.7	45.7–51.7	1,348,601
Roraima	430,939	25.0	22.3–27.9	107,735	48.6	45.0–52.2	209,436
Pará	5,971,477	26.2	23.2–29.3	1,564,527	45.2	41.8–48.7	2,699,108
Amapá	570,298	23.4	20.2–26.9	133,450	45.9	41.6–50.3	261,767
Tocantins	1,125,023	33.1	29.0–37.6	372,383	52.2	48.7–55.7	587,262
Maranhão	4,873,279	30.0	26.3–34.0	1,461,984	48.5	43.9–53.0	2,363,540
Piauí	2,383,425	32.7	29.4–36.1	779,380	53.0	49.6–56.3	1,263,215
Ceará	6,788,403	33.8	31.0–36.7	2,294,480	53.7	50.8–56.6	3,645,372
Rio Grande do Norte	2,632,403	33.2	30.2–36.3	873,958	52.9	49.7–56.1	1,392,541
Paraiba	2,984,647	33.4	30.6–36.3	996,872	49.0	46.0–51.9	1,462,477
Pernambuco	7,035,040	33.2	30.7–35.8	2,335,633	53.4	50.8–55.9	3,756,711
Alagoas	2,377,983	31.7	28.6–35.0	753,821	53.5	49.7–57.3	1,272,221
Sergipe	1,688,955	30.8	28.0–33.8	520,198	50.0	46.7–53.2	844,478
Bahia	11,044,986	30.3	26.8–34.1	3,346,631	48.9	44.8–53.0	5,400,998
Minas Gerais	16,425,183	35.6	33.1–38.2	5,847,365	55.1	52.0–58.2	9,050,276
Espírito Santo	3,047,439	31.5	27.6–35.6	959,943	48.1	43.6–52.7	1,465,818
Rio de Janeiro	13,419,464	36.0	33.8–38.1	4,831,007	55.8	53.6–58.0	7,488,061
São Paulo	35,414,776	35.6	33.7–37.4	12,607,660	58.2	56.2–60.2	20,611,400
Paraná	8,736,014	34.9	31.7–38.2	3,048,869	57.1	53.3–60.9	4,988,264
Santa Catarina	5,578,842	34.1	30.2–38.2	1,902,385	55.9	51.6–60.1	3,118,573
Rio Grande do Sul	8,902,263	39.5	36.8–42.3	3,516,394	58.4	55.6–61.1	5,198,922
Mato Grosso do Sul	2,045,881	34.7	31.6–37.8	709,921	57.6	54.5–60.7	1,178,427
Mato Grosso	2,543,642	31.9	28.9–35.1	811,422	54.8	51.9–57.6	1,393,916
Goiás	5,277,383	34.4	31.5–37.4	1,815,420	52.0	49.1–54.9	2,744,239
Distrito Federal	2,310,636	29.9	27.3–32.5	690,880	49.2	46.3–52.1	1,136,833

N at risk: number of adults (≥18 years) at risk of severe Covid-19

Criterion 1: age ≥ 65 years or diagnosis of cardiovascular disease, diabetes, chronic respiratory disease, hypertension, cancer (< 5 years of diagnosis), or stroke; Criterion 2: additionally obesity (BMI ≥ 30 kg/m^2^), current smoking, chronic kidney disease (diagnosis and under Hemodialysis, peritoneal dialysis, taking medication or did a kidney transplant), moderate to severe asthma (diagnosis and taking inhalers, aerosol or tablets)

## DISCUSSION

In this study, we estimated that a third (53 million) to over a half (86 million) of Brazilian adults present at least one risk factor for severe Covid-19. Our findings point to high prevalence of serious medical conditions in younger, but mostly, among older adults. Less educated adults present 2-fold higher prevalence of risk factors compared with university graduated. São Paulo, Rio de Janeiro, Minas Gerais and Rio Grande do Sul were the most vulnerable states in absolute and relative terms of adults at high-risk. Contrasts between South and Southeast vs North and Northeast regions might be due to different age structure, prevalence of health condition and/or access to medical diagnosis and care.

Estimating the proportion of the population at risk for severe Covid-19 within and across countries is key to improve prevention measures. However, to our knowledge, these estimates are still sparse worldwide. In the US, it was estimated that four in ten (37.6%) adults ≥ 18 years may be at high-risk of severe Covid-19^14^. During the pandemic, time is limited and hence the use of existing health information to support countries’ response is imperative. These findings and methods to identify high-risk settings may be useful to plan and manage prevention strategies in Brazil and other low- to middle-income settings with routinely collected data from population-based surveys, but limited testing capacity for SARS-CoV-2.

The understanding of risk factors for severe Covid-19 has so far supported the implementation of prevention strategies. It is interesting to note that non-communicable diseases such as cardiovascular disease, cancer, respiratory diseases, and diabetes, which accounts for most of deaths globally^15^, play a role on worsening the impact of the Covid-19 pandemic. Since isolation of infected cases and contact tracing alone will not likely suffice to control the pandemic^16^, countries have largely implemented social isolation measures. The combination of different interventions such as case isolation, social distancing of the entire population, household quarantine, school closure and, ultimately, complete lockdown is predicted to have significant impact on transmission^17^. Protecting the groups that are most at risk^18^, such as older adults and people with comorbidities, by widely and temporarily refraining from engaging in social contact, remains imperative. As knowledge on the clinical course of Covid-19 advances, the understanding of risk factors for severe disease will be improved, and so will the estimates of most-at-risk populations.

Our results have some limitations. Prevalence of risk factors for severe Covid-19 is likely underestimated due to self-reported medical diagnosis of comorbidities and smoking status. Underlying diseases have been associated with poorer prognosis among inpatients with Covid-19, but some people may have lower risk due to well-controlled blood pressure and serum glucose, for instance, which may have overestimated the proportion and number of adults at risk. Undiagnosed, asymptomatic diseases such as diabetes and hypertension are concerns, especially in low-income settings. This may partially explain differences of adults at risk between Brazilian states. Estimates considered the same weight for all risk factors assessed, which may not be applicable. Furthermore, other known risk factors for severe Covid-19 such as living in a nursing home or long-term care facility, and immunosuppression could not be captured in our study. Lastly, risk factors information date from 2013, the most recent representative, household-based health survey of Brazilian adults. The proportion of older adults has increased in Brazil in the past seven years, as well as the prevalence of obesity and other non-communicable diseases^19^, which may have underestimated our estimates. On the other hand, the prevalence of tobacco smoking has decreased, which may have overestimated the adults at risk of severe Covid-19.

In conclusion, proportion and total number of adults at risk of severe Covid-19 is high in Brazil, with wide variation across states and adult subgroups. These findings should be considered while designing and implementing prevention measures. We argue that these results support broad social isolation measures, particularly while testing capacity for SARS-CoV-2 is limited.
